# Anatomic rupture location, leukocyte levels and diabetes mellitus as factors influencing in-hospital mortality following percutaneous repair of post-infarction ventricular septal rupture: a single centre study

**DOI:** 10.3389/fcvm.2025.1612869

**Published:** 2025-10-09

**Authors:** Faisal Habib, Nadya Keumala Fitri, Tengku Winda Ardini, Ali Nafiah Nasution

**Affiliations:** ^1^ Department of Cardiology and Vascular Medicine, Adam Malik Hospital, Medan, Indonesia; ^2^ Faculty of Medicine, Universitas Sumatera Utara, Medan, Indonesia

**Keywords:** post-infarction ventricular septal rupture (PIVSR), percutaneous closure, mortality factors, in-hospital outcomes, long-term follow-up, risk factors, single center study, Indonesia

## Abstract

**Background:**

Post-infarction ventricular septal rupture (PIVSR) carries high mortality despite therapeutic advances. This study evaluates outcomes and mortality predictors in 22 PIVSR patients treated at H. Adam Malik Hospital, Medan, where percutaneous closure has become preferred due to surgical limitations.

**Methods:**

This single-center, retrospective cohort study analyzed 22 consecutive patients with post-infarction ventricular rupture (January 2022–May 2025), stratified by closure eligibility (*n* = 11 per group). Comparative analyses used independent *t*-tests (normal data), Mann–Whitney-*U* tests (non-normal), and Fisher's exact tests (categorical variables). Survival analysis employed Kaplan–Meier curves with log-rank testing. Effect sizes (mean differences, risk differences, odds ratios) are reported with 95% confidence intervals.

**Results:**

The non-closure group had higher leukocyte counts [14.9 ± 5.9 vs. 11.0 ± 4.0 × 10⁹/L, mean difference −3.9 (95% CI, −7.6 to −0.1) × 10^9^ /L; *P* = 0.045], greater diabetes prevalence [54.5% vs. 9.1%; risk difference −45% (−75 to −16); *P* = 0.032], and shorter pre-closure survival (Mean 6 ± 5 days vs. 9 ± 6 days; *P* < 0.001). Among closure patients, apical rupture predicted universal mortality [7/7 deaths vs. 0/4 with mid-ventricular ruptures; risk difference 75% (25–100); *P* = 0.024], while LVEF (*P* = 0.92) and complexity (*P* = 1.000) showed no association. Survival favored closure (log-rank *P* < 0.001).

**Conclusion:**

Percutaneous PIVSR closure improved survival, but outcomes depended on anatomic rupture location, with apical rupture exhibiting prevalent mortality in the closure group. Non-closure patients had shorter survival, higher leukocytes, and diabetes. While rupture location and systemic factors influenced results, further research is needed to explain these associations and optimize patient selection.

## Introduction

Post-infarction ventricular septal rupture (PIVSR) is a severe but rare complication of acute myocardial infarction (AMI). Even with timely reperfusion, its modern incidence is much less than 1% of AMI cases, with an overall mortality rate around 40%–60% (1–5). The severity of hemodynamic failure in PIVSR is primarily driven by the massive left-to-right shunt volume and resultant cardiogenic shock. This shunt drastically diminishes left ventricular output, leading to systemic hypoperfusion and the progression of cardiogenic shock (6). While pulmonary vascular resistance plays a limited role in the acute phase due to the rapid onset of volume and pressure overload preventing adaptive changes, the combination of PIVSR and cardiogenic shock still results in a documented mortality rate ranging from 20% to 87% (7). Given the high mortality and complex hemodynamic consequences of PIVSR, timely and effective management is critical to improving patient outcomes.

Historically, surgical repair has been considered the gold standard for treating PIVSR, particularly for large or complex ruptures, offering a higher success rate in closing the defect and a decreased likelihood of residual shunt (8, 9). However, surgery necessitates an open-chest procedure, carrying inherent risks of complications and a prolonged recovery, which can be prohibitive for high-risk patients (10) to address potential hemodynamic instability, device assistance is frequently warranted with percutaneous closure. Percutaneous closure, despite offering faster recovery and acute defect reduction, may not immediately resolve low cardiac output syndrome stemming from myocardial ischemia and pre-operative right ventricular overload (11–14).

Percutaneous closure, while offering a less invasive alternative to surgery, presents unique challenges including residual shunts, technical difficulties, and device-related complications that are inherently dependent on rupture anatomy (15, 16). Traditionally reserved for smaller defects (<15 mm) in elderly or high-surgical-risk patients, recent consensus papers advocate for delayed intervention whenever clinically feasible, regardless of treatment modality (17). The procedure typically employs femoral arterial access with a 6F sheath, utilizing either the antegrade method or the retrograde method, Aiming for the formation of an arteriovenous loop for device placement (18–20).

Despite technical advances, PIVSR maintains alarmingly high mortality rates. Critical prognostic factors include leukocytosis, cardiogenic shock, and declining LVEF (21). Underscoring the need for meticulous consideration of treatment selection, optimal timing, procedural methodology, post-interventional care. This study examines in-hospital and long-term outcomes of percutaneous PIVSR closure at our Indonesian tertiary center, providing real-world insights into this challenging intervention's feasibility and outcomes.

## Methods

Data were retrospectively acquired from the medical and electronic records of patients treated for PIVSR at our single center in Medan, Indonesia, between January 2022 and May 2025. All consecutive patients diagnosed with PIVSR at our center during the study period were included in the overall cohort analysis. Patients undergoing percutaneous closure comprised a subgroup analysis. The collected data encompassed patient demographics, clinical features, pre-procedural clinical condition, echocardiographic characteristics, procedural details, procedural complications, in-hospital outcomes, and vital status at follow-up.

The exclusion criteria included an incomplete medical record. Additionally, patients were considered for percutaneous intervention if they met the following criteria after initial stabilization: (1) hemodynamic stability (MAP ≥65 mmHg without escalating support), (2) evidence of adequate end-organ perfusion (lactate ≤4 mmol/L, urine output ≥0.5 ml/kg/hr), (3) no life-threatening arrhythmias within 24 h, and (4) consensus by the heart team that procedural risks were acceptable. The diagnosis of cardiogenic shock was established using the ESC definition for patients with acute coronary syndromes. Intra-aortic balloon pump (IABP) was the only mechanical circulatory support available and utilized, as extracorporeal membrane oxygenation (ECMO) was not accessible at our institution during the study period. For patients undergoing percutaneous PIVSR closure, only the first attempt at closure was considered in the analysis.

Normally distributed continuous data are presented as mean ± standard deviation (SD), while non-normally distributed data are reported as median (first and third quartiles). Categorical data are displayed as frequency (percentage). To evaluate differences between closure and non-closure, comparative analyses is employed. All variables were first assessed for normality using Shapiro–Wilk testing, with group comparisons performed using independent *t*-test (for normally distributed continuous variables, reported as mean ± SD with Cohen's d effect sizes) or Mann–Whitney *U* tests [for non-normal distributions, reported as median (IQR) with rank biserial correlation]. Categorical variables were analyzed using Fisher's exact tests (for sparse data with expected cell counts <5) or Pearson ×2 tests, with odds ratios calculated for binary outcomes. Within the closure-eligible subgroup (*n* = 11) we further stratified outcomes by infarct location, LVEF, and rupture complexity. Survival differences were assessed using Kaplan–Meier analysis. All statistical analyses were performed using RStudio version 4.4.2 (Posit Inc., Boston, MA).

## Result

### Study and patient characteristics

Between January 2022 and May 2025, 22 patients were diagnosed with postinfarction ventricular septal rupture (PIVSR) at the Cardiac Centre, H. Adam Malik Hospital in Medan, Indonesia. All patients underwent percutaneous coronary intervention (PCI) prior to PIVSR management, with a median interval of 3 days (IQR: 1–8 days) following infarction diagnosis. Of these, 11 underwent transcatheter closure (Closure Group), while the remaining 11 died before the procedure could be performed (*N*on-Closure Group). Although surgical repair remains the gold standard for PIVSR—often combined with CABG, aneurysmectomy, or valve intervention—our center increasingly adopted a primary percutaneous approach for selected patients during the study period (22). This shift was driven by several factors: the technical challenges of operating on acutely infarcted, friable myocardium; limited availability of specialized surgical adjuncts and hybrid capabilities; and the need for timely intervention in hemodynamically unstable patients. Following multidisciplinary evaluation, percutaneous closure—typically with concomitant revascularization—was deemed the most feasible and safer option for these high-risk cases, prioritizing procedural feasibility and patient safety within our institutional context.

**Figure 1:**
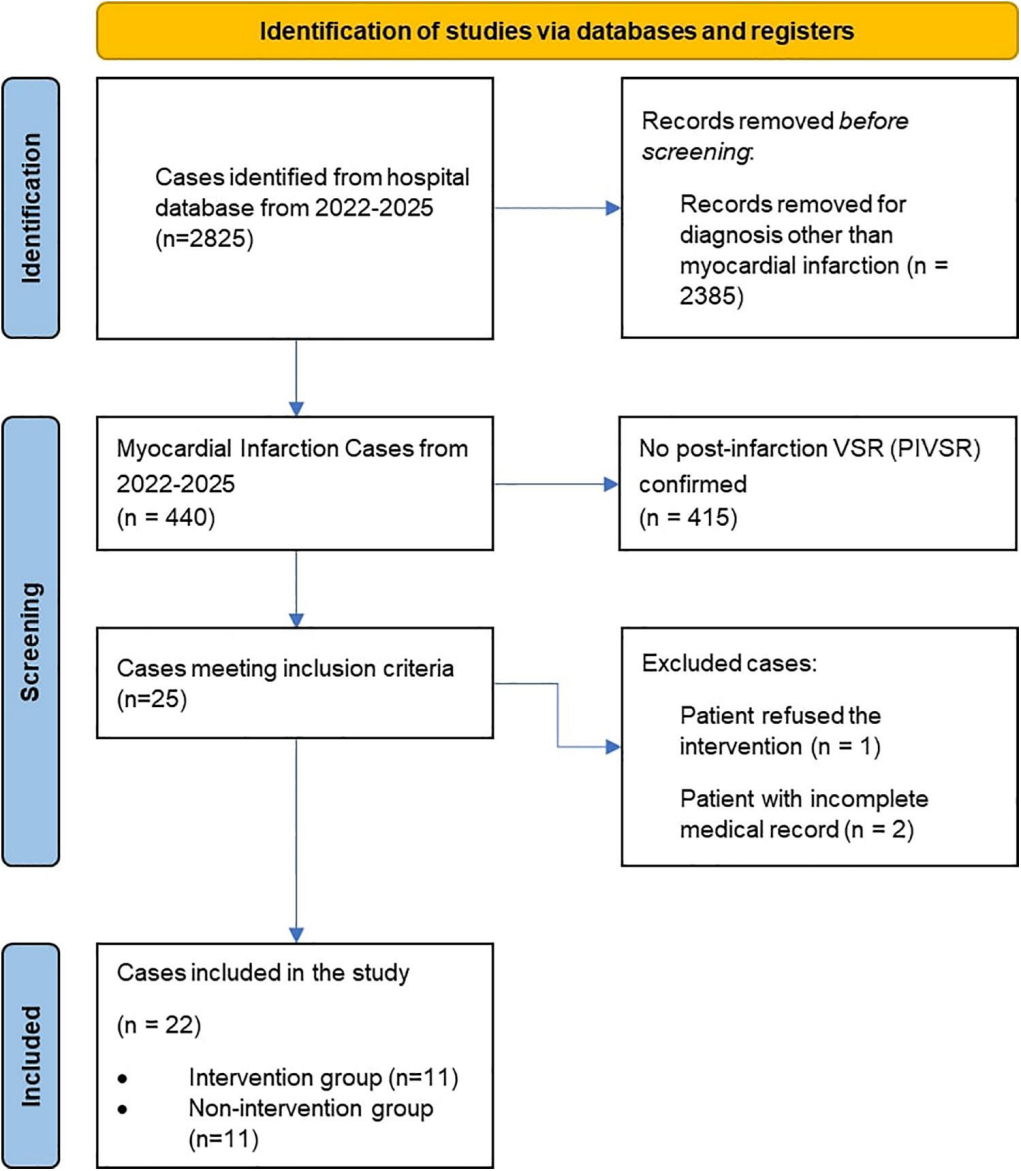
Flowchart of patient enrollment and exclusions.

The enrollment and exclusion of cases are detailed in Figure 1, of 22 eligible patients, 11 were allocated to each group. Baseline characteristics, in Table 1, were comparable between groups in terms of age (Closure Group: 60.0 ± 9.2 years; Non-Closure Group: 60 ± 12 years; *P* = 0.901) and sex distribution (36.4% vs. 54.5% female). However, the Non-Closure Group had a higher prevalence of diabetes mellitus (54.5% vs. 9.1%) and chronic kidney disease (45.5% vs. 9.1%). Both groups predominantly presented with anteroseptal infarction (72.7% vs. 81.8%) and left anterior descending (LAD) artery occlusion (63.6% vs. 90.9%). The Non-Closure Group exhibited more severe heart failure on admission, with higher Killip class 3–4 scores (36.4% and 27.3% vs. 0% and 9.1% in the Closure Group) and a trend toward higher leukocyte counts [14.9 ± 5.9 vs. 11.0 ± 4.0 × 10^9^ /L, mean difference −3.9 (95% CI, −7.6 to −0.1) × 10^9^ /L; *P* = 0.045].

**Table 1:** Patient demographic.

Group	Closure group (*n* = 11)	Non-closure group (*n* = 11)
Age, mean (SD)	60.0 (9.16)	60.36 (11.59)
Female sex, no. (%)	4 (36.4)	6 (54.5)
Diabetes mellitus, no. (%)	1 (9.1)	6 (54.5)
Hypertension, no. (%)	3 (27.3)	5 (45.5)
History of previous infarct, no. (%)	4 (36.4)	3 (27.3)
History of long-term smoking, no. (%)	6 (54.5)	4 (36.4)
Kidney disease, no (%)		
CKD (Chronic Kidney Disease)	1 (9.1)	5 (45.5)
AKI (Acute Kidney Injury)	7 (63.6)	4 (36.4)
Infarct territory, no. (%)		
Anterior	1 (9.1)	1 (9.1)
Antero septal	8 (72.7)	9 (81.8)
Antero-inferior	2 (18.2)	1 (9.1)
Vessel stenosis		
LAD (Left Anterior Descending)	7 (63.6)	10 (90.9)
LCx (Left Circumflex)	2 (18.2)	0
RCA (Right Coronary Artery)	2 (18.2)	1 (9.1)
Killip score, no. (%)		
1	2 (18.2)	3 (27.3)
2	8 (72.7)	4 (36.4)
3	0 (0)	1 (9.1)
4	1 (9.1)	3 (27.3)
Cardiogenic shock, no. (%)		
SCAI C	4 (36.4)	3 (27.3)
SCAI D	1 (9.1)	2 (18.2)
LV function, no. (%)		
>50%	2 (18.2)	3 (27.3)
30%–50%	7 (63.6)	7 (63.6)
<30%	2 (18.2)	1 (9.1)
Admission to closure/ pre closure death, mean days (SD)	9 (5.8)	6 (5)
Duration of symptoms, mean days (SD)	18 (3.1)	8 (4.5)
Leukocyte count upon admission, mean (SD)	10,998.2 (3,953.4)	14,867.3 (5,854.5)

**Figure 2:**
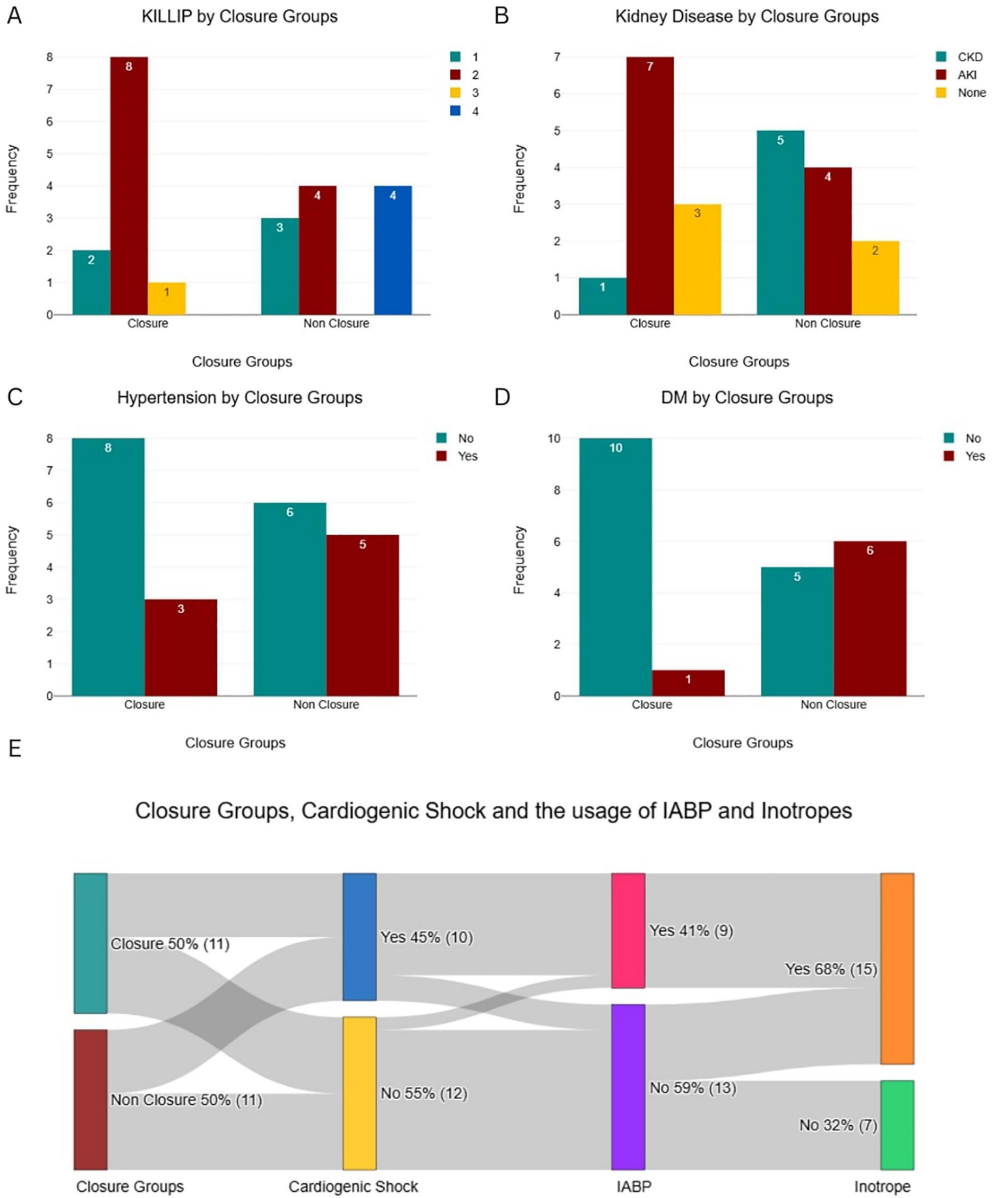
Baseline categorical characteristics and hemodynamic support utilization by patient group. **(A)** Killip Class distribution. **(B)** Prevalence of Kidney Disease. **(C)** Prevalence of Hypertension. **(D)** Prevalence of Diabetes Mellitus (DM). **(E)** Interrelationships between Closure Group, Cardiogenic Shock, and the usage of IABP and Inotropes.

**Figure 3:**
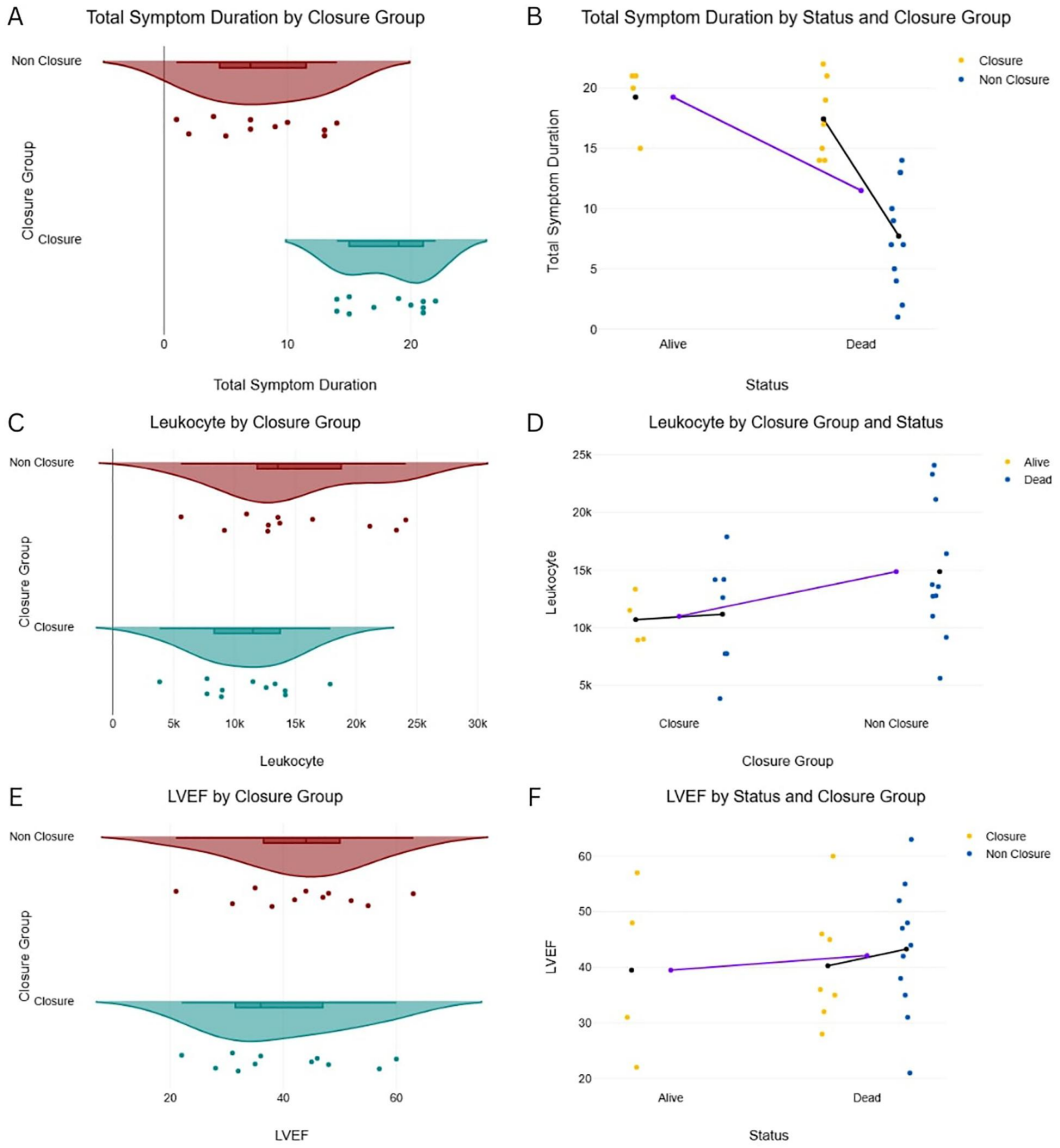
Total symptom duration, leukocyte count, LVEF by closure distribution of key continuous variables by patient group and mortality status. **(A)** Left Ventricular Ejection Fraction (LVEF) by Closure Group. **(B)** LVEF by Status and Closure Group. **(C)** Leukocyte count by Closure Group. **(D)** Leukocyte count by Closure Group and Status. **(E)** Total Symptom Duration by Closure Group. **(F)** Total Symptom Duration by Status and Closure Group.

Despite greater clinical instability in the Non-Closure Group, the Closure Group received more advanced hemodynamic support, including intra-aortic balloon pump (IABP) (54.5% vs. 27.3%) and inotropes (72.7% vs. 63.6%) (Figure 2), likely reflecting their prolonged survival and opportunity for stabilization prior to intervention. Symptom duration was significantly longer in the Closure Group (18 ± 3.1 vs. 8 ± 4.5 days; *P* = 0.571), while left ventricular function (LVEF 30%–50% in 63.6% of both groups) and cardiogenic shock severity (SCAI C/D: 45.5% vs. 45.5%) were similar (Figure 3).

### Percutaneous closure characteristics

**Figure 4:**
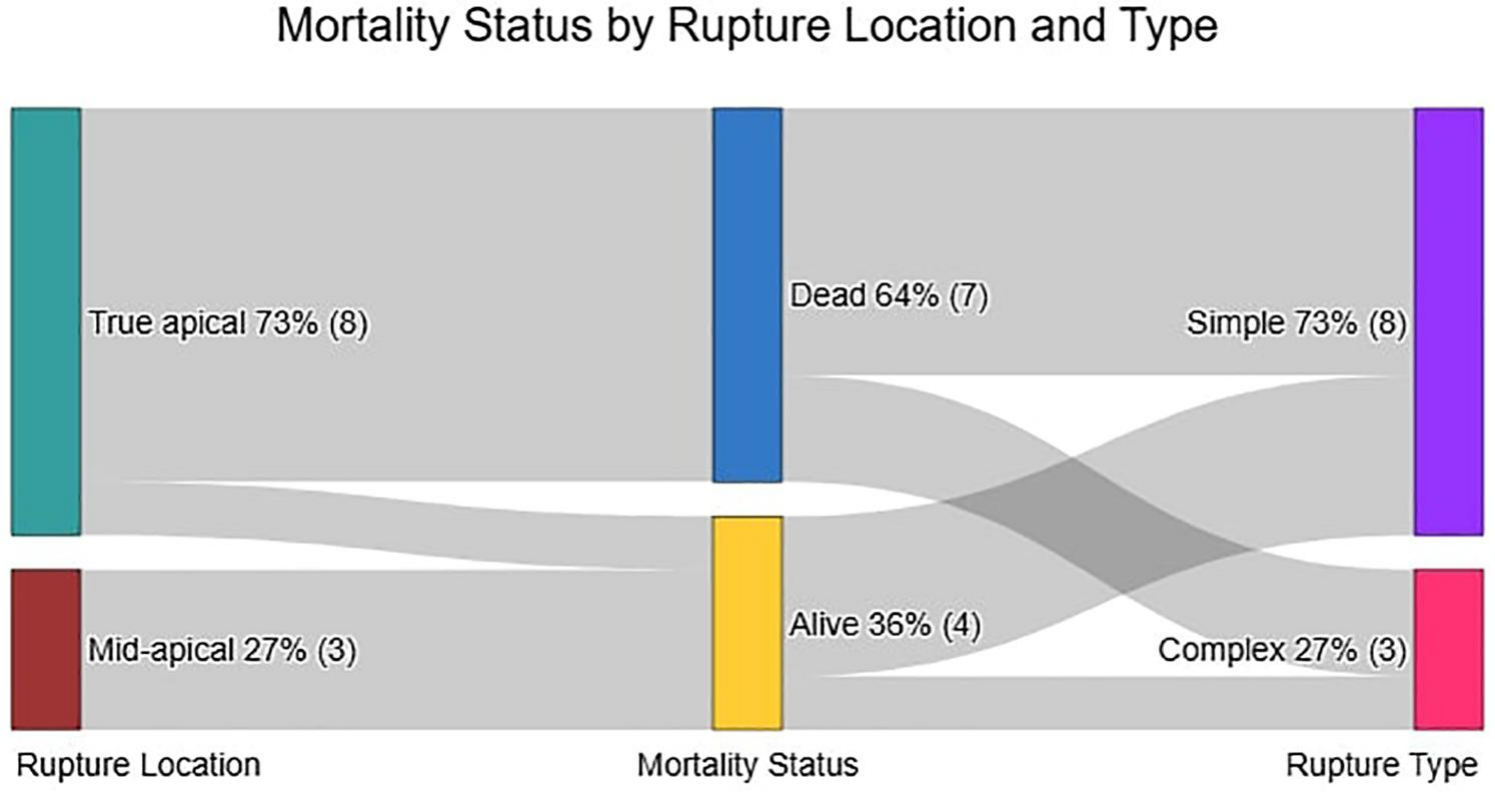
Relationship between rupture location, mortality Status, and rupture type in the closure group.

All 11 patients in the Closure Group underwent successful device implantation (Table 2). Ruptures were predominantly apical, with one case involving both apical and mid-muscular defects (Figure 4). Rupture sizes ranged from 5 to 27 mm (mean 13.3 mm). The most commonly used device was the Lepu Memopart ASD occluder (6 cases), followed by Occlutech ASD, mVSD, Lifetech KONAR MFO, and Lepu Memopart PDA occluders.

**Table 2:** Procedural characteristics.

Characteristics	*n* (%)
Rupture location, no. (%)	
True apical	8 (72.7)
Mid apical	3 (27.3)
Rupture type	
Simple	8 (72.7)
Complex	3 (27.3)
Rupture size, Median (Q1–Q3)	14 (8–16)
Device	
Occlutech ASD no. 18	2 (18.2)
Occlutech mVSD no. 22	1 (9.1)
KONAR-MF VSD Occluder no. 14–22	1 (9.1)
Lepu Memopart ASD no. 18–36	6 (54.5)
Lepu Memopart PDA Occluder no. 20	1 (9.1)
Immediate reduction in shunt	
Successful implantation with no central residual leakage	7 (63.6)
Successful implantation with central residual leakage	4 (36.4)

Post-procedural outcomes revealed although four had residual leakage, four patients were successfully discharged and seven deaths occurred -two immediately post-procedure and five at a median of 6 days (Q1–Q3: 2–11) (Table 3). Causes of death included sepsis (2), acute kidney injury (1), massive pleural effusion (1), and bleeding from iatrogenic thrombocytopenia (1). Among survivors (median follow-up 371 days, Q1–Q3: 193–540), all remained alive with no further complications, though they reported exertional dyspnea without syncope or recurrent chest pain.

**Table 3:** Intervention patient details.

No.	Age, Gender	Diagnosis	Defect diameter (TEE)	Onset to admission—admission to shunt days	Device	Methods	Prognosis	Discharge status
1	51, F	STEMI Killip IV, Single defect	8 × 9 mm	14–6	Occlutech ASD No. 18	Retrograde with arteriovenous loop	Successful implantation, minimal central residual leakage (-)	Alive
2	63, M	STEMI Killip II, Multiple defects	Defect I: 5 × 4 mm Defect II: 5 × 3 mm	16–5	Lifetech KONAR MFO No. 14–12	Antegrade with arteriovenous loop	Successful implantation, minimal central residual leakage (-). Flow from adjacent defect (+)	Alive
3	53, M	STEMI Killip II, Multiple defects	Defect I (16 × 8 mm), Defect II (8 × 4 mm) & Defect III (4 × 2 mm)	3–3	Occlutech mVSD No. 22	Retrograde with arteriovenous loop	Successful implantation, minimal central residual leakage (-)	Alive
4	52, M	STEMI Killip II, Single defect	11–14 mm, (Serpiginous)	7–14	Lepu Memopart ASD No. 18	Antegrade with arteriovenous loop	Successful implantation, minimal central residual leakage (-)	Alive
5	70, M	STEMI Killip II, Single defect	8 × 16 mm	18–1	Lepu Memopart ASD No. 20	Antegrade with arteriovenous loop	Successful implantation, minimal central residual leakage (-)	Death day 15 post operative, due to sepsis (WBC count: 16.290 /μl)
6	79, F	STEMI Killip II, Single defect	16–17 mm	10–7	Lepu Memopart ASD No. 21	Antegrade with arteriovenous loop	Successful implantation, minimal central residual leakage (+)	Death day 2 post operative, due to acute kidney injury (Cr: 1.03–>4.93)
7	59, F	STEMI Killip II, Single defect	12 × 9 mm	1–12	Lepu Memopart ASD No. 22	Antegrade with arteriovenous loop	Successful implantation, minimal central residual leakage (-)	Death day 9 post operative, due to massive pleural effusion + iatrogenic thrombocytopaenia
8	60, M	STEMI Killip I, Single defect	14–15 mm	7–7	Occlutech ASD No. 18	Retrograde with arteriovenous loop	Successful implantation, minimal central residual leakage (+)	Death day 2, post operative due to sepsis (WBC count: 3,850 /μl)
9	73, M	STEMI Killip I, Single defect	14 × 10 mm	1–11	Lepu Memopart ASD No. 22	Antegrade with arteriovenous loop	Successful implantation central residual leakage (+)	Death day 0 due to shock
10	54, M	STEMI Killip III, Single defect	16 × 27 mm	10–11	Lepu Memopart ASD No. 36	Antegrade with arteriovenous loop	Successful implantation central residual leakage (+)	Death day 1 due to shock
11	49, F	STEMI Killip III, Single defect	11 × 22 mm	3–11	Lepu Memopart ADO No. 20	Antegrade with arteriovenous loop	Successful implantation central residual leakage (+)	Death day 0 due to shock

### Statistical analysis

**Figure 5:**
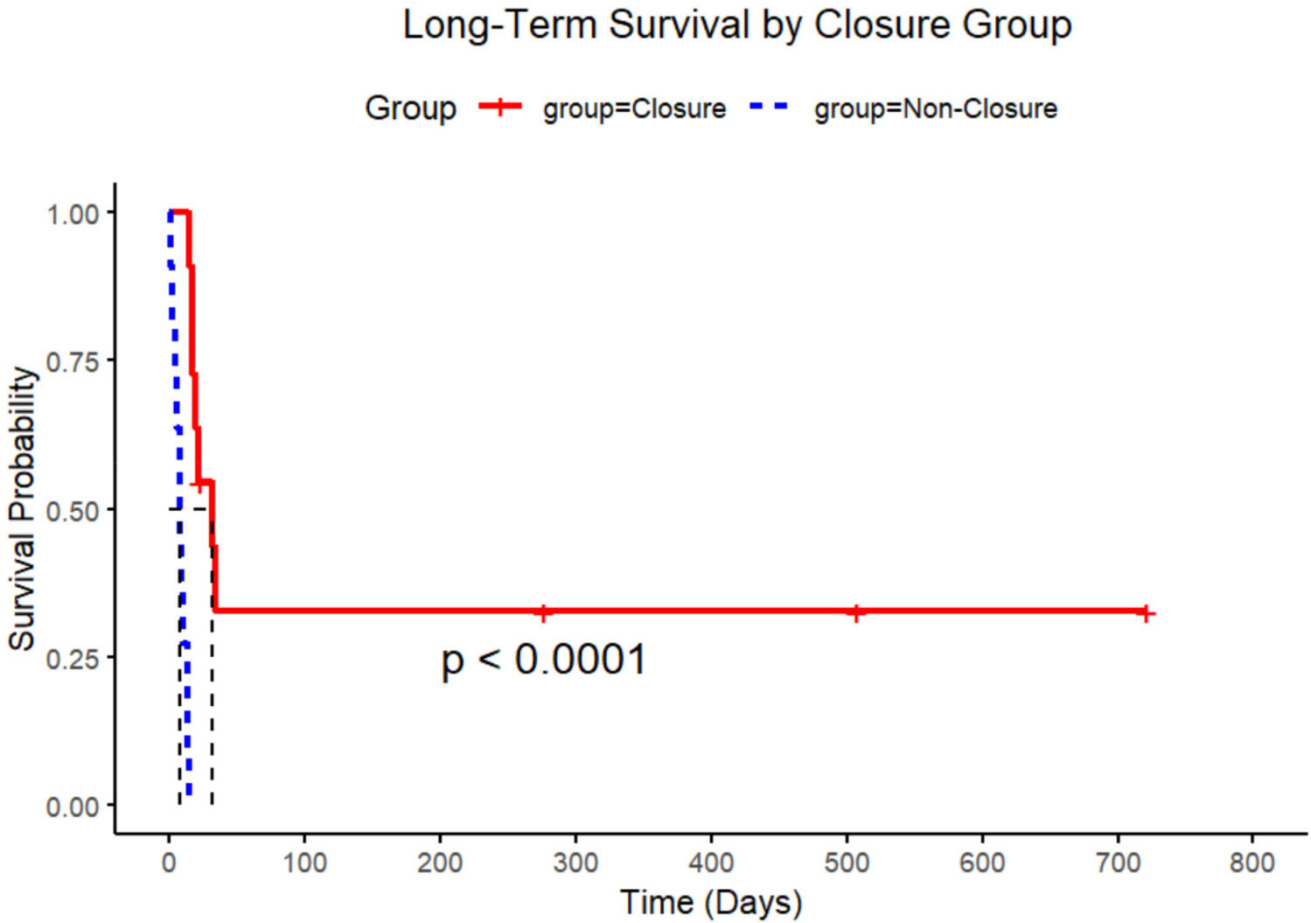
Kaplan–Meier survival curves by closure group. Extended survival probabilities over time. The statistical significance of the survival difference between groups is indicated by the *p*-value (*p* < 0.0001).

In this comparative analysis of 22 post-infarction patients (11 closure-eligible vs. 11 non-closure, Table 4), the non-closure group demonstrated significantly higher leukocyte counts [14.9 ± 5.9 vs. 11.0 ± 4.0 × 10⁹/L, mean difference −3.9 (95% CI, −7.6 to −0.1) × 10^9^ /L; *P* = 0.045] and diabetes prevalence [54.5% vs. 9.1%; risk difference −45% (−75 to −16); *P* *=* 0.032], along with shorter time of pre-closure survival (Mean 6 ± 5 days vs. 9 ± 6 days; *P* < 0.001). Among closure patients (Table 5), apical rupture location was universally fatal (7/7 deaths) compared to 100% survival in mid-ventricular ruptures (0/4 deaths). The odds of death were significantly higher for apical ruptures [Odds Ratio 38.2 (95% CI, 1.7 to ∞); *P* = 0.024]. Whereas neither LVEF (Mean 39.5 ± 15.9% vs. 40.3 ± 10.9%) in survivors vs. non-survivors; *P* *=* 0.92) nor rupture complexity (*P* *=* 1.000) predicted outcomes. Survival analysis confirmed superior outcomes in the closure group (log-rank *P* < 0.0001), with anatomic location emerging as the dominant prognostic factor over traditional risk markers (Figure 5).

**Table 4:** Significant comparative analysis of closure vs. Non-Closure Groups^a^.

Variable	Closure group (*N* = 11)	Non-closure group (*N* = 11)	Difference (95% CI)	*P* value
Laboratory results				
Leukocyte count (×10^9^ /L)	11.0 ± 4.0	14.9 ± 5.9	−3.9 (−7.6 to −0.1)	0.045
Comorbidities				
Diabetes mellitus—no. (%)	1 (9)	6 (55)	−45% (−75 to −16)	0.032
Temporal factors				
Time to admission (days)	9.0 (3.0–14.0)	6.0 (1.0–13.0)	3.0 (1.2–4.8)^b^	<0.001

^a^ Plus–minus values are means ± SD; IQR denotes interquartile range.

^b^ Median difference.

**Table 5:** Outcomes by survival Status in the closure subgroup (*N* = 11)^a^.

Variable	Alive (*n* = 4)	Dead (*n* = 7)	Effect size (95% CI)	*P* value
LVEF, %	39.5 ± 15.9	40.3 ± 10.9	−0.8 (−18.9 to 17.3)	0.92
Rupture location—no. (%)				0.024
Apical	1 (25)	7 (100)	Odds ratio: 38.2 (1.7 to **∞)**	
Mid-ventricular	3 (75)	0 (0)		
Rupture complexity—no. (%)				1.000
Simple	3 (75)	5 (71)	Odds ratio: 1.2 (0.1 to 19.6)	
Complex	1 (25)	2 (29)		

^a^ CI denotes confidence interval; LVEF, left ventricular ejection fraction. Data are mean ± SD or No. (%). *P* values from independent *t*-test (LVEF) or Fisher's exact test (categorical variables).

## Discussion

This study presents the outcomes of post-infarction ventricular septal rupture (PIVSR) treatment in a cohort of 22 patients at the Cardiac Centre, H. Adam Malik Hospital, Medan, Indonesia, from January 2022 to May 2025.

The Non-Closure group exhibited a higher prevalence of diabetes (54.5%), hypertension (45.5%), and chronic kidney disease (45.5%). While direct comparisons between the Closure and Non-Closure groups were limited by sample size, this trend suggests that baseline comorbidities may influence clinical trajectories. Notably, a higher leukocyte count on admission was independently associated with assignment to the Closure Group. This aligns with prior studies linking systemic inflammation (e.g., elevated WBC count or C-reactive protein) to poorer prognoses (5, 21, 23–25). Patients with lower inflammatory markers or better-controlled inflammatory responses may have been more likely to stabilize sufficiently for percutaneous intervention. Neither leukocytosis nor comorbidities like diabetes were exclusion criteria for closure. Instead, the heart team's decisions prioritized dynamic clinical stability (e.g., hemodynamic adequacy, end-organ perfusion) over baseline risk factors. The association of these variables with outcomes may reflect their role in exacerbating physiological stress—for example, infection-driven leukocytosis worsening shock or diabetes impairing microvascular recovery.

This, along with the known significant impact of comorbidities and prior infarction on mortality in PIVSR patients, ssuggests that patients with a less severe comorbidity burden were more likely to proceed with intervention (26, 27). This likely reflects their better overall clinical stability, a crucial factor within the inherently vulnerable context of PIVSR. The consistently high mortality rate in the non-closure group further emphasizes the critical nature of PIVSR and the urgent need for timely, effective intervention.

The prevalence of cardiogenic shock in both groups are equal with 45.5% highlights the severe hemodynamic compromise inherent to PIVSR, necessitating advanced supportive measures such as intra-aortic balloon pump (IABP) therapy and inotropes. While PIVSR frequently leads to unavoidable shock, especially with delayed intervention, the role of immediate percutaneous closure for achieving hemodynamic stability is nuanced. As noted in our introduction and recent literature, some patients may still experience low cardiac output syndrome post-closure despite defect closure, suggesting that acute myocardial injury can cause lasting dysfunction (28–30). Nevertheless, IABP therapy remains a recommended adjunctive treatment, as it can significantly reduce left-to-right shunt volume, increase systemic cardiac output, reduce afterload, improve coronary perfusion, and decrease myocardial oxygen consumption, thereby bridging patients to definitive therapy (31–33).

Transcatheter closure was successfully performed in all 9 patients, with defect sizes ranging from 9 to 15 mm. The predominance of apical defects aligns with existing literature on common rupture sites post-MI. The use of the Lepu Memopart ASD device in the majority of cases (6 cases) indicates its perceived suitability and ease of deployment in our experience. However, the presence of central residual leakage in 4 cases (36.4%) is higher in comparison to larger studies which reported reported lower rates (12%)—possibly due to hybrid rescue options. underscores the technical challenges associated with achieving complete closure (5). Regarding device innovation, the Occlutech PIVSD has been conceived with the aim of transforming the circular configuration into an ellipsoidal one, allowing for safe self-expansion into the rupture and better adaptation to the irregularly serpiginous rupture track (34).

Interestingly, our study found that rupture location, specifically a mid-apical rupture, was a statistically significant predictor of mortality status in the closure subgroup. This negative coefficient suggests that a mid-apical rupture location was associated with lower odds of mortality in patients undergoing percutaneous closure. This finding is less frequently emphasized in prior reports that primarily differentiate between anterior and posterior ruptures (35, 36). This novel observation suggests that apical ruptures may present distinct anatomical and hemodynamic characteristics that favor successful closure or contribute to better outcomes in this specific context. Further investigation into mortality differences based on precise rupture location, such as apical vs. mid-apical defects, remains a worthwhile pursuit for future research, beyond current broad classifications.

Within this study, the median time from diagnosis to closure and the median duration of the symptomatic period were both >7 days, reflecting a pragmatic approach often driven by the patient's clinical condition. Consistent with recent consensus papers advocating for a delayed approach whenever clinically feasible, our center's strategy is to delay intervention, timing it from the onset of the event (37). The unfortunate deaths of patients before intervention, particularly with a median time to death of 7 days after the event, highlight the severity and rapid progression of PIVSR. While our center's evolving strategy favored percutaneous approaches for selected cases, decisions regarding emergent surgical intervention for other patients were complex, often influenced by prohibitive surgical risk deemed by the heart team and resource limitations.

Despite technically successful device implantation, post-procedural mortality remained high (7/11 patients, 63.6%), primarily due to systemic complications including shock, sepsis, acute kidney injury (AKI), massive pleural effusion, and heparin-induced thrombocytopenia (HIT). These outcomes underscore that technical success often fails to translate into survival in this critically ill population. A representative case illustrates this challenge: one patient developed life-threatening massive pleural effusion (secondary to pneumonia and pulmonary edema) compounded by iatrogenic thrombocytopaenia, a condition commonly resulted by HIT (Heparin Induced Thrombocytopaenia)—an immune-mediated prothrombotic disorder caused by anti-PF4/heparin antibodies (38).

This created a management paradox, as heparin cessation (essential for HIT) conflicted with myocardial infarction treatment requirements. We addressed this with platelet transfusion while managing the effusion through drainage, diuretics, and antibiotics. Notably, AKI occurred despite standardized contrast use (100 cc Ultravist 370 mg) in all patients, suggesting its etiology extended beyond contrast nephropathy to reflect overall illness severity. In severe cases, hemodialysis was required, though with limited impact on the overall poor prognosis associated with multiorgan failure.

The median time to death was 6 days post-procedure, emphasizing the critical nature of the early post-procedural period. This aligns with existing literature, which consistently reports high overall mortality rates in PIVSR patients due to multi-organ failure and sepsis, even after successful defect closure (39, 40). The high post-procedural mortality in our small cohort, even with successful closure, underscores the critical need for improved patient selection, judicious timing of intervention, and optimized perioperative management to ultimately enhance outcomes in this challenging patient population. The growing use of transcatheter closure as a less invasive alternative to surgical repair has gained traction in recent years, with studies demonstrating comparable success rates and lower procedural morbidity making optimization of pre- and post-procedural care paramount (12, 41).

## Limitations

This study has several important limitations. First, the small sample size (*N* = 22 overall, *N* = 11 closures) restricts statistical power and generalizability compared to larger series like the Indian Heart Journal (IHJ) 2025 study (*N* = 142). While both studies identify systemic complications as key mortality drivers, our limited cohort precluded detection of additional predictors (e.g., right ventricular dysfunction) reported in their multicenter registry. Second, the retrospective, single-center design introduces selection bias and reflects outcomes specific to our resource-constrained setting, where percutaneous closure was the sole option, unlike the IHJ cohort's hybrid surgical-percutaneous approach. Despite these constraints, our findings provide insights into anatomical (e.g., mid-apical VSR survival benefit) and logistical challenges in under-resourced settings—a critical perspective absent in larger registries.

## Conclusion

Our study demonstrates that percutaneous closure of PIVSR is associated with improved survival, though outcomes vary significantly by anatomic rupture location. Apical rupture emerged as a high-risk predictor of mortality in the closure group, while non-closure patients exhibited shorter pre-closure survival, elevated leukocyte levels, and higher diabetes prevalence. These findings highlight the importance of rupture location and systemic factors in determining outcomes. However, further research is needed to elucidate the underlying mechanisms and to validate whether risk-stratified selection, technical precision, and post-procedural care directly influence survival.

## Data Availability

The raw data supporting the conclusions of this article will be made available by the authors, without undue reservation.
